# Travaux D’Electrotherapie Gynécologique

**Published:** 1896-02

**Authors:** 


TRAVAUX D'ELECTROTHERAPIE GYNÉCOLOGIQUE: Archives Semestrielles d'Electrotherapie Gynécologique Fondées et Publiées par Le Dr. G. Apostolí, Vice-President de la Société Française d'Electrothérapie, etc. Paris: Société d'Editions Scientifiques. 4 Rue Antoine-Dubois, 1894. Prix, 12 francs.

This volume contains a collection or compendium of all the most important *mêmoires* upon the subject of the electrotherapeutics of gynæcology in all the different languages of Europe, gathered together by and edited by Dr. Apostoli. This branch of medicine is claimed by the Editor to be wholly of French origin, and to have had for its father Dr. A. Tripier.

There are contributions on the treatment of uterine tumors by Drs. Thomas Keith and Skene Keith, of course translated from English into French; remarks on electrical treatment for uterine troubles by Sir T. Spencer Wells; proceedings of the Medico-Chirurgical Society of Brighton on electrical treatment; remarks of Professor Playfair before the British Medical Association; remarks of Dr. Ephraim Cutter, of New York; of Dr. Lawson Tait; of John Inglis Parsons, and of many others. Numerous clinical cases are given and a few drawings of apparatus.

